# Macrophage-associated pro-inflammatory state in human islets from obese individuals

**DOI:** 10.1038/s41387-019-0103-z

**Published:** 2019-12-02

**Authors:** Wei He, Ting Yuan, Kathrin Maedler

**Affiliations:** Islet Biology Laboratory, Centre for Biomolecular Interactions, University of Bremen, Bremen, Germany

**Keywords:** Type 2 diabetes, Obesity

## Abstract

Obesity is associated with inflammatory macrophages in insulin responsive tissues and the resulting inflammatory response is a major contributor to insulin resistance. In insulin-producing pancreatic islets, the intra-islet accumulation of macrophages is observed in patients of type 2 diabetes (T2D), but such has not been investigated in obese individuals. Here, we show that pro-inflammatory cytokines (IL-1β, IL-6, and TNF), anti-inflammatory cytokines (IL-10 and TGF-β) and macrophage polarization markers (CD11c, CD163, and NOS2) were expressed in isolated human islets from non-diabetic donors. Clodronate-mediated depletion of resident macrophages revealed expression of *IL1B* and *IL10* mostly from macrophages, while *IL6, TNF*, and *TGFB1* came largely from a non-macrophage origin in human islets. *NOS2* expression came exclusively from non-macrophage cells in non-obese individuals, while it originated also from macrophages in obese donors. Macrophage marker expression of *CD68*, *CD163*, and *ITGAX was* unchanged in islets of non-obese control and obese cohorts. In contrast, *IL1B* and *NOS2* were significantly increased in islets from obese, compared to non-obese individuals, implying a more inflammatory macrophage phenotype in islets in obesity. Our study shows elevated macrophage-associated inflammation in human islets in obesity, which could be an initiating factor to the pro-inflammatory intra-islet milieu and contribute to the higher susceptibility to T2D in obese individuals.

## Introduction

Tissue macrophages reside in pancreatic islets as well as in almost all other tissues from very early development. These islet-associated macrophages maintain tissue homeostasis and support normal tissue function. However, under inflammatory triggers, they become deleterious to pancreatic β-cells^1^ and are, therefore, a potential target for the therapy of diabetes. Obesity is a major risk factor for the development of type 2 diabetes (T2D), as the associated chronic, low-grade, sterile inflammation contributes to both insulin resistance and finally, β-cell failure. Studies in rodents reveal that obesity-induced diabetes is associated with increased numbers of macrophages in pancreatic islets with more pro-inflammatory phenotypes^1^. In this current study, we aimed to identify whether such pro-inflammatory islet state also occurs in human obesity by defining islet macrophage-associated genes related to inflammation and macrophage polarization in isolated islets from non-obese and obese individuals.

## Materials and methods

Human islets were isolated from pancreases of non-diabetic organ donors. Human islet culture, depletion of islet macrophages, preparation of islet-conditioned macrophages/media, and gene expression analyses were performed as previously described^2,3^. Isolations from 28 donors were collected between 2014 and 2018, and classified into control lean to overweight non-obese (BMI < 30, *n* = 16) and obese (BMI > 30, *n* = 12) cohorts (donor details in ESM Table 1). Statistical significance was tested using Student’s *t-test* for single comparison or two-way ANOVA for multiple comparisons. Correlation analyses were performed using Spearman’s correlation.

## Results and discussion

Recently, we showed that islet resident macrophages are the major source of interleukin (IL)-1β but not of IL-6 and tumour necrosis factor (TNF) in human islets. As we had stimulated inflammatory conditions by TLR-2/-4 activation in this previous study^2^, we wanted to confirm such macrophage-dependency of cytokine expression under physiological, as well as diabetes-prone conditions.

**Fig. 1:**
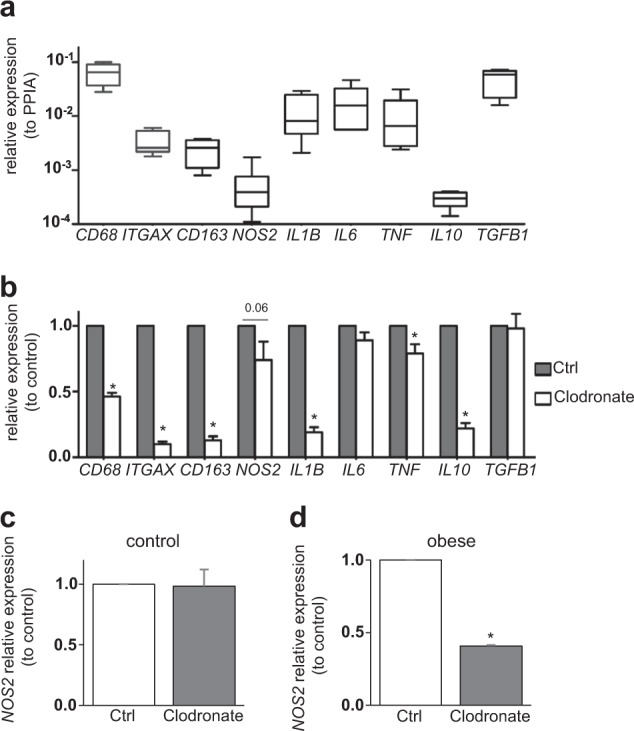
Macrophage-dependent and -independent inflammation marker expression within isolated human islets. **a** mRNA expression levels relative to housekeeping gene (cyclophilin A, *PPIA*) from human isolated islets. **b–d** Comparative gene expression analysis of clodronate liposome (1 mg/ml for 48 h) and vehicle liposome (Ctrl)-treated human islets. Separate analysis of *NOS2* expression from control (**c** BMI < 30, *n* = 4) and obese donors (**d**, BMI > 30, *n* = 3). **a**, **b** *n* = 5–7. Data are means ± SEM *p < 0.05 Ctrl vs. clodronate.

Major pro-inflammatory cytokines (IL-1β, IL-6, and TNF), anti-inflammatory cytokines (IL-10 and TGF-β) as well as macrophage polarization markers (CD11c, CD163, and NOS2) were expressed in isolated human islets from non-diabetic donors regardless of their BMI at basal conditions (Fig. 1a). Among them, *TGFB1* (gene for TGF-β) and *CD68* had a particularly high expression, while *IL10* and *NOS2* had very low but constant expression in all donors (Fig. 1a). Depletion of resident macrophage by clodronate treatment was shown to be successful by the significant reduction of the general macrophage markers *CD68*, pro-inflammatory macrophage marker *ITGAX* (gene for CD11c) and anti-inflammatory macrophage marker *CD163* (reduced by 54%, 90%, 87%, respectively; Fig. 1b). In these macrophage-depleted human islets*, IL1B* and *IL10* were largely deprived (by 81%, 78%, respectively; Fig. 1b), suggesting their macrophage-dependent expression. *TNF* was only partially but significantly reduced by 21% (Fig. 1b), implying not only macrophages but also other islet cells as the major source of TNF expression. Basal expression of *IL6* and *TGFB1* was unchanged by macrophage depletion (Fig. 1b), indicating their non-macrophage origin in human islets.

*NOS2* only showed a slight and insignificant reduction by 26% compared to the untreated group, with the highest variation among all donors (Fig. 1b). Separate analysis of non-obese (BMI < 30) and obese donors (BMI > 30) revealed no difference in islets from non-obese donors (Fig. 1c), while in islets from obese donors, *NOS2* was reduced by 59% in macrophage-depleted islets vs. control (Fig. 1d). This result is in line with a basal *NOS2* expression in β-cells^4^, and implies a macrophage-derived *NOS2* expression induced in obesity.

Although tissue macrophages are usually the major source of cytokines, they do not seem to fit into this paradigm in human pancreatic islets. Macrophages are indeed the main contributor of IL-1β and IL-10 expression in islets. In contrast, IL-6 and TGF-β are not macrophage derived, and they also barely contribute to the intra-islet TNF production. Most likely, the islet microenvironment shapes a peculiar tag to its resident macrophages. A cytokine expression profile present already under physiological conditions suggests the existence of intra-islet triggers to a sensitive inflammatory program, which impacts β-cell function, survival and proliferation^5–7^. Indeed, a low-grade basal tissue cytokine expression doesn’t necessarily mean an inflammatory response. Instead, many cytokines support important tissue functions, e.g., acute IL-1β exposure promotes survival and insulin secretion of β-cells, and IL-10 maintains insulin sensitivity of adipocytes^8–10^.

**Fig. 2:**
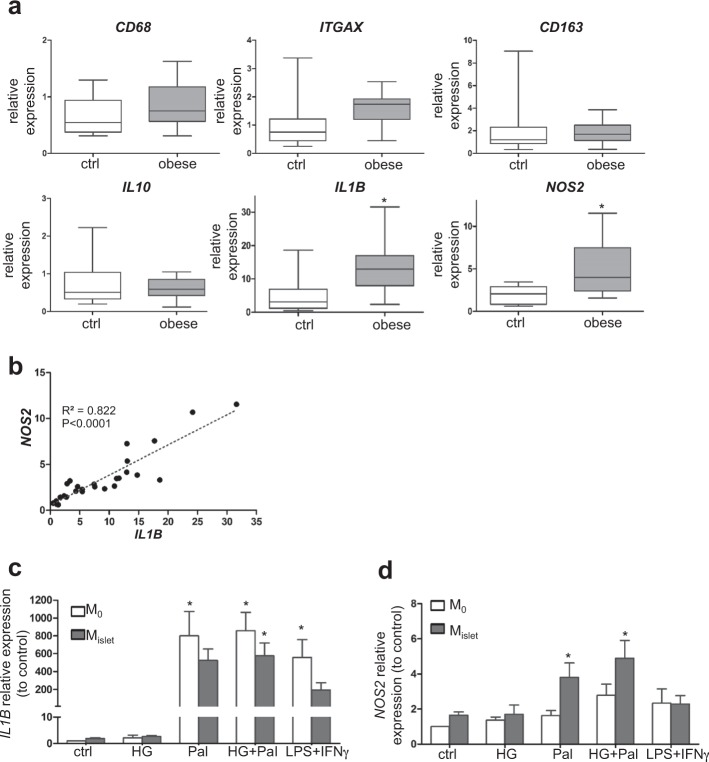
*IL1B* and *NOS2* expression in human macrophages under gluco-/lipotoxicity. **a** Comparative gene expression analysis of isolated islets from control (BMI < 30, *n* = 16) and obese (BMI > 30, *n* = 12) cohorts. Data are means with whiskers. **b** Correlation of *IL1B* and *NOS2* expression among all donors (*n* = 28). **c**, **d** *IL1B* and *NOS2* expression in islet-conditioned human macrophages (M_islet_) and control non-conditioned macrophages (M_0_) treated with 22.2 mM glucose (HG), 0.5 mM palmitate (Pal), combined HG and Pal, or combined 100 ng/ml LPS and 1000 U/ml IFNγ for 24 h. Data are means ± SEM, *n* = 4. *p < 0.05 control vs. treatment or control vs. obese cohort.

Obesity is associated with the accumulation of pro-inflammatory macrophages in fat and liver^11^. Increased macrophages in islets in response to long-term high-fat diet feeding contribute to the intra-islet inflammation and the loss of insulin secretion in obese mice^7^. However, the situation in human pancreatic islets has not been clearly studied. Therefore, we next investigated whether such pro-inflammatory environment also exists in human islets and included more human islet isolations from non-diabetic donors for gene expression analyses of islet macrophage-dependent genes identified above. 28 donors were divided into islets from non-obese (BMI < 30, *n* = 16) and obese (BMI > 30, *n* = 12) cohorts (donor details in ESM Table 1). Unexpectedly and in contrast to a previous in-depth mouse study^7^, the general macrophage marker *CD68*, the M2 macrophage marker *CD163* and even the marker for pro-inflammatory macrophages *ITGAX were* not significantly changed between control and obese cohorts (Fig. 2a), disfavoring macrophage accumulation in human islets of obese individuals. However, the latters showed enormous variations among non-obese donors (Fig. 2a). Also, anti-inflammatory cytokine *IL10* was unchanged between control and obese cohorts. Indeed, islets from T2D patients display more macrophages^12^, which may suggest a delayed macrophage accumulation process in human islets compared to the diet-induced obesity mouse model^7^.

In contrast, *IL1B* and *NOS2* were significantly increased in the obese, compared to the non-obese cohort (Fig. 2a) and both are highly correlated in all donors (Fig. 2b), implying a more inflammatory macrophage phenotype in obesity. In line with these data, *IL1B* and *NOS2*, both commonly considered as markers for inflammatory macrophages, were highly upregulated under severe T2D states^4,5^.

In order to mimic islet macrophage-derived obesity-induced *IL1B* and *NOS2* expression in vitro, we used islet-conditioned macrophages, which were differentiated from blood monocytes under islet-conditioned medium^3^. The combination of 22.2 mM glucose (HG) and 0.5 mM palmitate (Pal) was added to the culture to represent the gluco-/lipotoxic milieu in vitro, mimicking the result of a chronic western diet with high content in glucose and fat, being the major risk factor for obesity in modern society. 24-hour treatment induced *IL1B* expression in both control (M_0_, non-conditioned) and islet-conditioned (M_islet_) macrophages (Fig. 2c), while palmitate alone induced *IL1B* expression in M_0_ but high glucose had no effect.

Palmitate alone or combined with HG induced *NOS2* expression in islet-conditioned but not in control macrophages (Fig. 2c), while combined lipopolysaccharide/interferon-gamma (LPS/IFNγ) induced *IL-1B* (as expected) but no *NOS2* expression in either macrophages. This suggests the existence of islet-derived factors to specifically facilitate *NOS2* induction upon palmitate/high-glucose treatment.

Overall, the upregulation of *IL1B* and *NOS2* in islets of obese donors and in response to a gluco-lipotoxic milieu in islet-conditioned macrophages indicates a pro-inflammatory phenotype within pancreatic islets associated with obesity.

As human β-cells have a very low basal expression of *NOS2*^4^, further studies are required to verify the elevated *NOS2* expression in islet macrophages during obesity. As an indirect support, palmitate alone or in combination with high-glucose concentrations induced *NOS2* expression in islet-conditioned, but not in un-conditioned human macrophages, underlining the necessity of both an gluco-/lipotoxic milieu (as a result of a chronic western diet) and the islet microenvironment for pro-inflammatory islet macrophages. This is further supported by the phenomenon that such effect is absent in the classical pro-inflammatory LPS/IFNγ condition. Indeed, in a number of different islet isolations from lean, overweight and obese organ donors, only islets from obese donors displayed macrophage-derived *NOS2*. This may be a critical point for islet inflammation during gluco-/lipotoxicity and obesity, as elevated *NOS2* levels are also present in T2D islets^4^.

Unlike NOS2, intra-islet IL-1β expression is almost exclusively dependent on macrophages, also true for TLR-2/4-triggered IL-1β production^2^. Chronic exposure of high-level IL-1β induces β-cell failure, whereas acute or low-level IL-1β promotes β-cell function and survival^5,8,10^. Hence, it would be more conceivable that obesity-induced islet IL-1β production initially contributes to the compensatory functional expansion of β-cell in response to the increased insulin demand, which is consistent with β-cell proliferation induced by islet macrophages in obese mice^7^. However, long-term functional overload will trigger β-cell failure, which is a consensus of T2D progression.

This study shows macrophage-associated inflammation in human islets in obesity. Owing to the very low number of macrophages in human islets (0.5–0.7 cells/islet)^12^, quantitative analyses for this study were only possible on the messenger RNA (mRNA) level. Improved single-cell sequencing techniques would provide a future path to further characterize these immune cells and more importantly, delineate their functions in the context of obesity and in the process of diabetogenesis.

## Supplementary information

Suppl.Table
