# Supplementary material for: Macrophage-associated pro-inflammatory state in human islets from obese individuals
Source: Nutr Diabetes. 2019 Dec 2;9:36. doi: 10.1038/s41387-019-0103-z (PMC6885511; doi:10.1038/s41387-019-0103-z)
Supplement: Supplementary file 1 — Suppl.Table [file 41387_2019_103_MOESM1_ESM.pdf]

Supplementary Table 1: Human islet donor characteristics

non-obese cohort	AGE	BMI	GENDER
1	31	23.8	M
2	23	24.5	F
3	16	21.8	M
4	57	29.4	F
5	51	22.3	M
6	65	23	M
7	57	26.1	M
8	57	23.1	F
9	48	26	F
10	46	25	F
11	55	23.5	M
12	50	21.7	M
13	32	19	F
14	48	25	M
15	50	19.6	M
16	42	20.2	F
**MEAN**	**45.5**	**23.38**	
SEM	3.37	0.67	
**obese cohort**			
1	25	36.9	F
2	45	32.9	M
3	43	31.7	M
4	38	32	M
5	24	31	M
6	40	32.8	F
7	36	31	F
8	56	33.4	M
9	53	32.8	M
10	54	40.6	M
11	38	34.4	F
12	35	35.2	M
**MEAN**	**40.58**	**33.72**	
SEM	2.99	0.8	
